# A Mobile Intervention to Link Young Female Entertainment Workers in Cambodia to Health and Gender-Based Violence Services: Randomized Controlled Trial

**DOI:** 10.2196/27696

**Published:** 2022-01-04

**Authors:** Carinne Brody, Pheak Chhoun, Sovannary Tuot, Anne E Fehrenbacher, Alexander Moran, Dallas Swendeman, Siyan Yi

**Affiliations:** 1 Public Health Program Touro University California Vallejo, CA United States; 2 KHANA Center for Population Health Research Phnom Penh Cambodia; 3 Department of Community and Global Health Graduate School of Medicine The University of Tokyo Tokyo Japan; 4 Department of Psychiatry and Biobehavioral Sciences Semel Institute University of California Los Angeles Los Angeles, CA United States; 5 Department of Epidemiology University of California Los Angeles Los Angeles, CA United States; 6 Saw Swee Hock School of Public Health National University of Singapore and National University Health System Singapore Singapore

**Keywords:** mHealth, female sex workers, HIV, sexually transmitted infection, linkage to services, sexual and reproductive health, gender-based violence, low- and middle-income countries

## Abstract

**Background:**

Female entertainment workers (FEWs) in Cambodia experience a greater prevalence of human immunodeficiency virus (HIV), other sexually transmitted infections (STIs), psychological distress, substance abuse, and gender-based violence (GBV) than the general female population. Reaching FEWs with health education and linking them to services has been difficult because of their hidden and stigmatized status.

**Objective:**

This study evaluated the efficacy of the Mobile Link intervention in improving FEWs’ health by engaging and connecting them to existing HIV, sexual and reproductive health, and GBV services.

**Methods:**

A randomized controlled trial was conducted between March 2018 and June 2019 in the capital city and 3 other provinces in Cambodia. FEWs in the intervention arm received automated twice-weekly Short Message Service messages and voice messages with health information and direct links to outreach workers. The control group received the existing standard care, including free HIV and STI counseling and testing and a toll-free helpline staffed by trained counselors. We used a stratified random sampling method to select participants from 5 study sites in the 4 selected provinces. Initially, we randomly selected 600 participants from a list of 4000 FEWs by age group (18-24 and 25-30 years) and study site using a random number generator and enrolled them in person. The primary outcome measures included self-reported HIV and STI testing, condom use, and contraceptive use assessed through a face-to-face structured interview. We also measured secondary outcomes, including contact with outreach workers, escorted referral service use, forced drinking, and GBV experiences. Intervention effects were modeled using repeated measures, multilevel mixed-effects logistic regression.

**Results:**

A total of 1118 participants were recruited and enrolled in the study. We included 218 FEWs in the intervention arm and 170 FEWs in the control arm in the per protocol analyses after removing 730 dropouts. Evidence of positive intervention effects was detected for the following secondary outcomes: contacting an outreach worker (at 30 weeks: adjusted odds ratio [AOR] 3.29, 95% CI 1.28-8.47), receiving an escorted referral (at 30 weeks: AOR 2.86, 95% CI 1.09-7.52; at 60 weeks: AOR 8.15, 95% CI 1.65-40.25), and never being forced to drink at work (at 60 weeks: AOR 3.95, 95% CI 1.62-9.60). Over time, no significant differences between intervention and control groups were observed for any primary outcomes in the fully adjusted models.

**Conclusions:**

The Mobile Link intervention effectively connected FEWs with outreach workers and escorted referrals but did not show an effect on primary outcomes. Reduced forced drinking at work was also significantly more extensive in the intervention group than in the control group. Longer-term messaging may increase access to services and impact FEWs’ health outcomes in the future.

**Trial Registration:**

Clinicaltrials.gov NCT03117842; https://clinicaltrials.gov/ct2/show/NCT03117842

**International Registered Report Identifier (IRRID):**

RR2-10.1186/s13063-018-2614-7

## Introduction

Female entertainment and sex workers experience a greater prevalence of human immunodeficiency virus (HIV), other sexually transmitted infections (STIs) [1,2], psychological distress [3], substance abuse [3,4], and gender-based violence (GBV) [1,5] than the general female population. These health burdens are intensified by social and structural factors, including poverty, gender inequality, discrimination, and stigmatization [1,2]. In Cambodia, these health trends and associations hold for female entertainment workers (FEWs) employed at establishments such as karaoke bars, restaurants, beer gardens, and massage parlors [6,7]. Approximately one-third of FEWs exchange sex to supplement their income [8]. Transactional sex puts FEWs at high risk for adverse health outcomes, threatening their psychological, physical, and family health [7,8].

FEWs in Cambodia often come from poor and rural families, originally migrating to cities at a young age in hopes of earning higher wages to send to their families [7-9]. FEWs have an HIV prevalence of nearly 10% and low HIV and STI testing rates and service seeking [10]. They also have low rates of modern contraceptive use and high rates of induced abortion [8,11] and are frequent victims of GBV [7,12]. The rates are higher among FEWs younger than 30 years than among the older groups [10-12].

FEWs face barriers to accessing health services. As an HIV key population in Cambodia, they are eligible to receive free health services provided by nongovernment organizations (NGOs). However, many do not seek services due to stigma and discrimination [10]. The population’s health risk profile is further exacerbated by Cambodia’s 2008 Law on the Suppression of Human Trafficking and Sexual Exploitation, which bans prostitution. Research indicates a strong association between sex work criminalization and sex workers’ increased risk for HIV and STIs, sexual and physical violence, and unprotected sex [13]. In Cambodia, outlawing prostitution has amplified the stigma against FEWs and deterred this population from carrying condoms, which the police use as evidence of sex work. The law also creates mistrust of the police among FEWs, which prevents them from reporting instances of GBV to the police, who frequently raid entertainment establishments and arrest FEWs [14,15]. Additionally, the law is associated with a substantial increase in the number of FEWs as more women move from brothels to entertainment venues, where they can continue to exchange sex [6,7]. In 2008, an estimated 13,000 FEWs were reported in Cambodia; that number had risen to approximately 40,000 by 2018 [16].

Health interventions using mobile phones, referred to as mobile health (mHealth), present a viable solution for connecting hard-to-reach, stigmatized, and criminalized populations, such as FEWs, to health services. In recent years, mHealth has received widespread attention due to its applicability in low-resource settings. mHealth has been used effectively in low- and middle-income countries to collect and report community health data [17], disperse health education information [17], raise health awareness [18], and conduct routine check-ins with patients and trigger follow-ups by nurses [19]. However, knowledge gaps persist in mHealth research. Few mHealth interventions have been rigorously evaluated. Much of the existing literature comprises small pilot studies lacking established health indicators and generalizability [20-22]. Additionally, there is a deficiency in mHealth research on interventions targeted toward behavior change and sexual and reproductive health (SRH) [21].

The Mobile Link intervention is an mHealth project aiming to engage and connect young FEWs in Cambodia to existing prevention, care, and treatment services using the automated Short Message Service (SMS) messages and voice messages (VMs). The Mobile Link intervention is based on behavior change theories and years of formative research. The intervention aims to reduce FEWs’ risk behaviors and increase HIV, STI, SRH, and GBV service utilization. The project’s details are described in the formative study and protocol papers, with an overview provided below [23,24]. This study aims to evaluate the efficacy of the Mobile Link intervention in engaging FEWs; connecting them to existing HIV, SRH, and GBV services; and ultimately improving their health. It strives to fill existing knowledge gaps in mHealth research and provide information about a hard-to-reach, high-risk, and understudied population.

## Methods

### Trial Design and Settings

The Mobile Link intervention study is a 2-arm multisite 60-week randomized controlled trial (RCT). The trial was conducted in 2 sites in Phnom Penh and 1 site each in Banteay Meanchey, Battambang, and Siem Reap. These provinces were selected because of substantial populations of FEWs and high HIV burdens.

### Participants

The intervention’s participant inclusion criteria included (1) being in the age group of 18-30 years; (2) self-identifying as a FEW; (3) working at an entertainment venue in the study sites; (4) being currently sexually active, defined as having engaged in oral, vaginal, or anal sex in the past 3 months; (5) owning a mobile phone; (6) knowing how to retrieve VMs or retrieve and read SMS messages; (7) willing to receive 2 SMS messages/VMs per week for 1 year; (8) providing written informed consent; and (9) agreeing to a follow-up visit after 6 and 12 months.

### Randomization

We based our sample size on information from our recent study, which found that 352 of 667 (52.8%) FEWs underwent at least 1 HIV test in the past 6 months [25]. We calculated a sample size that would be able to detect an increase of 13% in HIV testing as a result of the Mobile Link intervention. The sample size was 600 based on a significance level of .05, with 80% power and accounting for 28% attrition. Field workers developed a list of more than 4000 FEWs from the 5 study sites. FEWs in the list were categorized by site and age group (18-24 and 25-30 years old). At each site, 120 FEWs were randomly selected using a random number generator: 60 (50%) in the age group of 18-24 years and 60 (50%) in the age group of 25-30 years. Furthermore, half of the selected FEWs (30 [50%] in the age group of 18-24 years and 30 [50%] in the age group of 25-30 years) were randomly assigned to the intervention arm and the other half to the control arm. Therefore, 60 participants from each arm from each of the 5 sites finally added up to 300 FEWs in the intervention and 300 FEWs in the control arm for a study total of 600 participants.

### Recruitment

All participants were recruited in-person at the 5 study sites by trained Mobile Link lay community health workers. Community health workers provided verbal information to FEWs regarding Mobile Link’s details because of low literacy rates in this population. Eligible FEWs signed the informed consent form and provided community health workers with mobile numbers for all of their subscriber identification module (SIM) cards and indicated which SIM they used most often. Recruited FEWs were assigned a unique identification number to protect their privacy and blind the researchers from their treatment arm assignment.

At midline, the data collection team recruited additional study participants to replace those who were lost to follow-up by randomly selecting FEWs from the same site and age group on the master list, excluding those who had ever been selected. These replacement participants got an opportunity to participate in the second 30 weeks of the intervention period. We considered participants having at least 2 survey assessments to be active participants in the study.

### Patient and Public Involvement

This clinical trial was developed after months of iterative qualitative data collection with FEWs, including 27 focus group discussions (FGDs), 9 in-depth interviews (IDIs), and 2 validation workshops. During this period, participants gave researchers guidance and feedback on the design and delivery of the intervention and recruitment and data collection procedures. Dissemination activities involved a validation workshop, where we presented and discussed findings from the formative studies with representatives of FEWs and key stakeholders, a midterm review workshop, and 2 final findings dissemination workshops.

### Intervention

The Mobile Link intervention was informed by behavior change theories and extensive formative research. The intervention provided FEWs with information, resources, and reminders. By utilizing an SMS/VM platform, these services were provided in a convenient, accessible, inexpensive, and confidential manner. Therefore, we theorized that this delivery mechanism would improve FEWs’ knowledge of existing resources, risks, risk behaviors, and positive attitudes related to these topics. Increasing knowledge and positive attitudes would contribute to skill acquisition and a positive behavior change.

We conducted a series of formative research activities using participatory methods to create appropriate and relevant health-related messages for FEWs and inform the intervention’s development. The formative research process occurred over 6 months. We collected data through FGDs, IDIs, and key informant interviews (KIIs) with venue- and non-venue-based FEWs, in addition to outreach workers and field staff routinely working with this population [23]. Findings from the formative research revealed that FEWs are generally knowledgeable about HIV and STI prevention and transmission. However, they face many structural barriers to optimal health, such as pressure to drink alcohol at work and complicated dynamics of negotiating condom use with clients in a criminalized environment [25,26]. Furthermore, we found that many FEWs face barriers to accessing medical care and services due to stigma, discrimination, and mistreatment from health care workers.

We developed the message-based intervention with the support of local partners InSTEDD iLab and the Women’s Media Center (WMC). InSTEDD developed a mobile platform for interactive message delivery and data management using an open source software program. The WMC helped translate messages into Khmer and tailor the contents to be specific, relevant, and engaging, given the cultural context. Example messages included can be found in a previously published paper [23].

After the development, the intervention underwent a 4-week pilot in which 50 FEWs from each study site were randomly selected. The purpose of the pilot was to test whether the platform functioned well with the intervention design and whether the intervention was feasible and acceptable for the participants and other key stakeholders.

The central components of the Mobile Link intervention were the SMS messages and VMs containing health information and referral linkage information to health services and resources. From the formative research process, 180 messages were designed covering 10 health themes identified as the most important by participants. The health themes covered the following topics: cervical cancer, contraception, general health information, HIV and STI transmission and prevention, miscarriage, pregnancy, alcohol use at work, pregnancy termination, hygiene and vaginal health, and GBV. A message was delivered twice a week for 10 weeks, and the message from each topic area was repeated every 10 weeks for 60 weeks. The health messages were framed using rights-based and health promotion frameworks. Participants could choose to receive the messages via SMS or in VM form that worked with simple and smartphone devices. Those who chose the SMS message option could further personalize their choice by selecting Khmer characters or Romanized Khmer. Each health topic message was followed by a message providing FEWs with the option to be linked to an outreach worker for free. Participants who selected this option were called by Mobile Link’s staff, who would provide individualized information over the telephone or face to face and, if needed, would escort the participant to services.

The controls for this study received the existing standard care. Standard care included face-to-face counseling, free HIV and STI testing and condoms, and clinic and hotline phone numbers with a toll-free helpline for clients staffed by trained counselors. The group did not receive the health-related SMS/VM component. However, they received a “check-in” SMS message or VM between baseline and midline and another between midline and endline to stay in touch with the participants and remind them of the interview appointments.

### Outcomes and Measures

The primary outcome measures of the Mobile Link intervention were (1) HIV testing, (2) STI testing when experiencing symptoms, (3) contraceptive use, (4) always using condoms with nonpaying partners, and (5) always using condoms with paying partners. The secondary outcome measures were (1) contact with outreach workers, (2) utilization of escorted referrals, (3) forced drinking at work, and (4) responses to GBV and GBV acceptance.

The primary and secondary outcomes were tracked and measured using self-reported data from the baseline, midline, and endline surveys. The survey questionnaires contained items on demographics and background history; entertainment work; sexual behaviors; condom use self-efficacy; HIV risk perception, testing, and treatment; STI testing and treatment; contraception use and pregnancy; GBV and inequity; substance abuse; psychological distress; linkage to health services; and exposure to the Mobile Link intervention. The questionnaires contained approximately 100 questions, which were either dichotomous (eg, yes/no), categorical (eg, type of contraception method used), ordinal (eg, always, frequently, sometimes, and never), or a ratio (eg, number of years working in entertainment venues). The questionnaires were adapted from validated questionnaires used in our previous research on FEWs in Cambodia. The questionnaires were created in English, translated to Khmer, and back-translated to English. The Khmer questionnaires were validated via a pilot test of 15 FEWs with similar characteristics to the intervention participants, who were later excluded from the main surveys.

### Data Collection

The data collection process involved a face-to-face baseline, midline, and endline questionnaire survey. The questionnaires were administered in person in Khmer by Mobile Link’s female field researchers using the open source Kobo Toolbox software installed on Android-operating tablets. Before data collection, the field researchers underwent a 2-day training in which the questionnaire was also pretested. Field staff connected FEWs to field researchers by making appointments with FEWs, following up the appointments, and guiding FEWs to predetermined interview locations. We conducted the baseline survey before the start of the intervention (March 2018), the midline survey at 6 months after baseline (November 2018), and the endline survey at 12 months after baseline (June 2019). The questionnaires took approximately 25-30 minutes to complete, and the field researchers were blind to the FEWs treatment arm assignment to reduce the possibility of bias.

### Ethical Considerations

The Mobile Link intervention engaged community and public health stakeholders to ensure that the study incorporates the best practices and strong ethical standards. Due to the sensitive nature of HIV, SRH, and GBV topics presented in the surveys and questionnaires, additional steps were taken to ensure participants’ safety and well-being. First, all data collectors received training related to asking sensitive questions. Second, upon obtaining informed consent, community health workers disclosed information, making clear the sensitive topics discussed in the data collection process. Third, participants were offered escorted referrals to counseling services and provided with services upon request. Participants could be connected to services in the event of an adverse outcome through the SMS/VM platform. In addition, participants could leave the study at any time. Furthermore, participants’ identities were kept confidential and stored securely in password-protected files. Coded identifiers were given to participants after obtaining informed consent. No participants’ personal identifiers were used in analyses or report writing. Participants received US $5 for compensation of time and transportation reimbursement for their participation. This study was approved by the National Ethics Committee for Health Research (NECHR; no. 142NECHR) within the Ministry of Health in Cambodia and the Touro College Institutional Review Board (no. PH-0117).

### Statistical Analyses

STATA/SE 15.1 (College Station, TX, USA) was used for statistical analyses. We tabulated participants’ baseline characteristics and distributions of primary and secondary outcome variables for the intervention versus the control arm for the analytic sample (participants with at least baseline and 30-week observation) using frequencies and proportions for categorical variables and means and SDs for continuous variables. These characteristics were compared by group using tests of association, including Pearson chi-square tests of homogeneity for categorical variables and Student *t* tests for continuous variables to ensure the balance between the study arms. We conducted both crude and cluster-adjusted pooled tests of association to account for clustering within workplace venues. Participant characteristics were then compared for the analytic sample (n=388) versus the non-analytic sample (n=733) to assess significant differences within and between groups for those retained in the study per protocol for at least 2 survey assessments (ie, analytic sample) versus those lost to follow up after the baseline assessment (ie, non-analytic sample).

Intervention effects were assessed using multilevel mixed-effects logistic regression to model all binary outcomes accounting for within-subject correlation from taking repeated measures on the same participants over time (2-level models with observations nested within individuals). Clustered standard errors were computed to account for the similarity of characteristics and behaviors among participants in the same venues and any possible contamination between study arms. Separate models were conducted for each primary and secondary outcome. Model fit was assessed for each outcome using the Akaike information criterion (AIC) and the Bayesian information criterion (BIC).

Predictors in each simple unadjusted 2-level mixed-effects logistic regression model included group, time, and group-by-time interaction terms. Intervention effects for each outcome were determined by group-by-time interaction terms at endline with a significant *P*<.05. Significant interactions indicating intervention effects were graphed using the *marginsplot* command. Midline effects (effects at 30 weeks) are displayed in the figures but not in the tables. For the fully adjusted primary and secondary outcome models, the following covariates were included to control alternative explanations: entertainment job venue type, province, cohabitation, age, and education. For primary outcomes, contact with outreach workers in the past 6 months was also included as a covariate to assess the impact of linkage support on HIV and STI testing, contraceptive use, and condom use.

As a sensitivity analysis, we used intention-to-treat (ITT) principles for modeling primary and secondary outcomes with all participants (n=1121), according to the arm to which they were assigned, and then compared to the results for each outcome from the per protocol modeling with the analytic sample. Per protocol analyses were undertaken to assess the intervention’s impact among those who actively participated in the study. Participants lost to follow-up after baseline, resulting in missing outcome data at 6 months, were considered non-users. ITT and per protocol results were consistent for all outcomes regarding the direction, strength, and significance of associations. As such, only the per protocol results are presented in the tables for ease of interpretation.

### Protocol Adaptations

There are several protocol deviations to note. The original protocol called for a 12-month (52-week) trial. However, due to high dropout rates at the midterm, we extended the trial to 60 weeks to recruit and enroll more participants who would have the chance to be exposed to the intervention for at least 30 weeks.

We did not anticipate the level of loss to follow-up that occurred and, therefore, did not have a plan in place for replacement recruitment in our original protocol. We decided to recruit replacement participants at midline by randomly selecting FEWs from our master list from the same venue and age group. In our analyses, we defined exposure as having had at least 30 weeks of exposure to the intervention.

Another deviation occurred in our group assignment plan. Initially, we planned to randomize at the entertainment venue level to conduct a cluster RCT. Before the implementation, we changed our trial design to randomize at the individual level due to the high level of movement of FEWs between venues. As a result, we modeled intervention effects using the individual rather than the venue as the analysis unit. We computed clustered standard errors based on the venue rather than including the venue as a level in the mixed-effects outcome models. Finally, we included the venue type (eg, karaoke bar, beer garden, other venues) as a covariate in all our models.

We planned to send out weekly survey questions to intervention participants on various health topics in our study protocol. During intervention development, we heard from pilot participants that they felt reluctant to provide that type of information through the phone. We were also concerned about message fatigue, privacy, and literacy and decided to omit that part of the intervention.

Finally, in our protocol, we stated that we would present an ITT analysis. Because the ITT and per protocol findings were the same, we decided to present the per protocol analysis for ease of interpretation.

## Results

**Figure 1:**
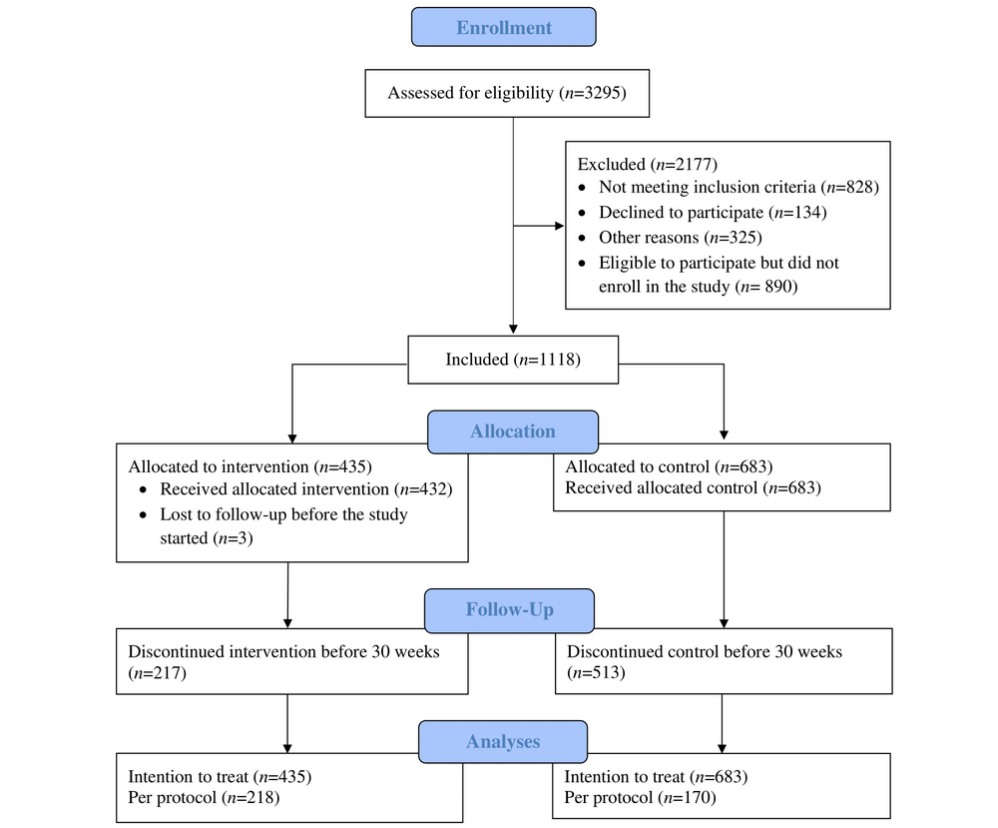
Participant flow diagram.

The study’s participant flow diagram is depicted in Figure 1. Before the intervention started, 3295 FEWs were assessed for eligibility, of whom 828 (25.13%) FEWs did not meet the eligibility criteria, 134 (4.07%) declined to participate, 3 (<0.01%) were lost to follow-up following randomization, and 325 (9.86%) were excluded because of other reasons. Of the included FEWs (2008/3295, 60.94%), 1118 (55.68%) were randomly selected for intervention randomization, of which 435 (38.91%) were allocated to the intervention group and 683 (61.09%) to the control group. By the end of 30 weeks, 217 of 435 (49.9%) FEWs in the intervention and 513 of 683 (75.1%) FEWs in the control group discontinued the study and were replaced. We included 435 (100%) FEWs in the intervention and 683 (100%) FEWs in the control group in intention-to-treat analyses and 218 of 435 (50.1%) FEWs in the intervention and 170 of 683 (24.9%) FEWs in the control group in the per protocol analyses.

Table 1 displays characteristics of the sample stratified by intervention versus control group with crude and cluster-adjusted pooled association tests. When accounting for clustering by venue, there were no differences in sample characteristics by group at baseline except a significantly lower proportion of currently married participants in the intervention than in the control group (42/218 [19.3%] vs 48/170 [28.2%], *P*=.05). There were crude differences identified by province and entertainment job venue type when not accounting for clustering within venues. The intervention group had a higher proportion of participants from Battambang than the control group (35/218 [16.1%] vs 15/170 [8.8%], *P*=.04). There were marginal but not significant crude differences between the proportions of intervention versus control participants in each of the other 3 provinces. The intervention group had a higher proportion of participants working in karaoke bars (146/218 [66.9%] vs 93/170 [54.7%], *P*=.01). In contrast, the control group had a higher proportion of participants working in beer gardens (26/218 [11.9%] vs 37/170 [21.7%], *P*=.01). All other characteristics were successfully matched between arms, with no statistically significant differences between intervention and control groups.

We compared the characteristics of the analytic (retained) versus non-analytic (lost to follow-up) samples and identified a significant baseline difference in being forced to drink at work. Participants in the analytic sample were more likely to believe that something can be done if a person experiences abuse and to report ever being forced to drink at work. No other baseline differences were identified between analytic and non-analytic samples.

**Table 1:** Baseline characteristics of female entertainment workers in the analytic sample of the Mobile Link study by intervention and control arms (N=388).

Baseline characteristics		Intervention group (n=218), n (%)	Control group (n=170), n (%)
**Demographics**			
	Age in years, mean (SD)	24.7 (3.8)	24.5 (4.0)
	Years of schooling attained, mean (SD)	6.2 (3.0)	6.3 (3.0)
**Marital status**			
	Currently married^a^	42 (19.3)	48 (28.2)
	Previously married (widowed, divorced, or separated)	81 (37.2)	55 (32.4)
	Never married	95 (43.6)	67 (39.4)
**Province**			
	Phnom Penh	86 (39.4)	51 (30.0)
	Battambang^b^	35 (16.1)	15 (8.8)
	Banteay Meanchey	52 (23.8)	55 (32.4)
	Siem Reap	45 (20.6)	49 (28.8)
Poor as a child (Multidimensional Childhood Poverty Scale score ≥ 3)		190 (87.2)	145 (85.3)
Weekly income in USD, mean (SD)		277.0 (206.2)	272.4 (167.0)
**Entertainment job venue type**			
	Karaoke bar^b^	146 (67.0)	93 (54.7)
	Beer garden^b^	26 (11.9)	37 (21.8)
	Other (eg, massage parlor, dance club, or freelance in streets/parks)	46 (21.1)	40 (23.5)
Had sex with partner in exchange for money or gifts, past 3 months (intervention n=146 [66.9%], control n=170 [100%])		60 (41.1)	40 (31.5)
**Type of message received**			
	SMS^c^ message	99 (45.4)	64 (37.6)
	VM^d^	119 (54.6)	106 (62.4)
**Primary health outcomes**			
	Tested for HIV^e^ in the past 6 months (intervention n=176 [80.7%], control n=150 [88.2%])	113 (64.2)	95 (63.3)
	Tested for STIs^f^ when most recently showed symptoms (intervention n=112 [51.4%], control n=71 [41.8%])	27 (24.1)	19 (26.8)
	Uses modern contraceptive to prevent pregnancy	68 (31.2)	64 (37.6)
	Always uses condom with nonpaying partners	43 (71.1)	31 (77.5)
	Always uses condom with paying clients (intervention n=60 [27.5%], control n=40 [23.5%])	23 (22.6)	17 (15.4)
**Frequency of forced drinking at work**			
	Never	123 (56.4)	111 (65.3)
	Less than monthly	16 (7.3)	13 (7.6)
	Monthly	28 (12.8)	15 (8.8)
	Weekly	51 (23.4)	31 (18.2)
Gender-Based Violence Acceptance Scale score (intervention n=26 [11.9%], control n=74 [43.5%]) (range 0-16), mean (SD)		4.4 (3.7)	4.5 (3.2)
Believes you cannot do anything if you or someone you know experiences physical or sexual abuse		60 (27.5)	47 (27.6)
Outreach worker contact only, past 6 months		20 (9.2)	13 (7.7)
**Number of times contacting outreach worker in the past 6 months**			
	Never	20 (46.5)	21 (58.3)
	Once	11 (25.6)	6 (16.7)
	2-4 times	11 (25.6)	8 (22.2)
	5 or more times	1 (2.3)	1 (2.8)
**Received escorted referral**			
	Received escorted referral for HIV/STI (intervention n=23 [10.6%], control n=23 [13.5%])	7 (30.4)	4 (17.4)
	Received escorted referral for vaginal health (intervention n=23 [10.6%], control n=23 [13.5%])	14 (60.9)	16 (69.6)

^a^Indicates a significant difference between intervention and control arms at baseline in a cluster-adjusted test of association by venue (*P*<.05).

^b^Indicates a significant difference between intervention and control arms at baseline in a crude test of association (*P*<.05).

^c^SMS: Short Message Service.

^d^VM: voice message.

^e^HIV: human immunodeficiency virus.

^f^STI: sexually transmitted infection.

Table 2 shows intervention effects at endline on primary outcomes in unadjusted and adjusted models (effects at 60 weeks). There was a statistically significant improvement in the frequency of self-reported condom use with nonpaying partners in the control group than in the intervention group in the unadjusted model (effects at 60 weeks: OR 0.26, 95% CI 0.08-0.83). In the intervention group, the proportion of FEWs who reported always using condoms with nonpaying partners was 22.6% (23/102) at baseline, 16.4% (19/116) at midline, and 17.2% (17/99) at endline. Among the control group, 15.5% (17/110) reported this condom use behavior at baseline, 24.8% (30/121) at midline, and 29% (11/38) at endline. However, no significant differences between intervention and control groups over time were observed for any primary outcomes in the fully adjusted models.

**Table 2:** Intervention effects on primary outcomes in the analytic sample in the unadjusted and adjusted models (N=989). Adjusted models included venue type, province, cohabitation, age, education, and outreach worker contact.

Primary outcomes	OR^a^ (95% CI)	AOR^b^ (95% CI)
Tested for HIV^c^, past 6 months (1=yes, 0=no, n=887 [89.7%])	0.45 (0.18-1.13)	0.40 (0.16-1.04)
Tested for STIs^d^, most recent symptoms (1=yes, 0=no, n=394 [39.8%])	1.36 (0.25-7.50)	1.20 (0.21-7.00)
Uses modern contraceptive to prevent pregnancy (1=yes, 0=no, n=989 [100%])	1.06 (0.37-3.02)	0.99 (0.35-2.77)
Always uses condom with nonpaying partners (1=yes, 0=no, n=586 [59.3%])	0.26 (0.08-0.83)^e^	0.50 (0.16-1.58)
Always uses condom with paying clients (1=yes, 0=no, n=242 [24.5%])	1.77 (0.14-22.74)	1.17 (0.08-17.64)

^a^OR: odds ratio.

^b^AOR: adjusted odds ratio.

^c^HIV: human immunodeficiency virus.

^d^STI: sexually transmitted infection.

^e^Indicates a significant difference between intervention and control arms at endline (*P*<.05).

As shown in Table 3, secondary outcomes with significant intervention effects at endline in unadjusted models included contacting an outreach worker (effects at 60 weeks: OR 3.31, 95% CI 1.06-10.33), receiving an escorted referral (effects at 60 weeks: OR 9.51, 95% CI 2.06-43.95), and never being forced to drink at work (effects at 60 weeks: OR 4.28, 95% CI 1.72-10.65). Statistically significant evidence for positive intervention effects at endline was detected for the following secondary outcomes in the fully adjusted models: receiving an escorted referral (effects at 60 weeks: AOR 8.15, 95% CI 1.65-40.25) and never being forced to drink at work (effects at 60 weeks: AOR 3.95, 95% CI 1.62-9.60). Significant midline effects were also detected for contacting an outreach worker (effects at 30 weeks: AOR 3.29, 95% CI 1.28-8.47) and receiving an escorted referral (effects at 30 weeks: AOR 2.86, 95% CI 1.09-7.52). This effect was not observed at endline for contacting an outreach worker in the adjusted model.

**Table 3:** Intervention effects on secondary outcomes in the analytic sample in the unadjusted and adjusted models (N=989). Adjusted models included venue type, province, cohabitation, age, and education.

Secondary outcome	OR^a^ (95% CI)	AOR^b^ (95% CI)
Outreach worker contact, past 6 months (1=yes, 0=no, n=989 [100%])	3.31 (1.06-10.33)^c^	2.82 (0.93-8.55)
Escorted referral, past 6 months (1= yes, 0=no, n=989 [100%])	9.51 (2.06-43.95)^d^	8.15 (1.65-40.25)^d^
Forced drinking at work, past 3 months (1=never, 0=ever, n=989 [100%])	4.28 (1.72-10.65)^d^	3.95 (1.62-9.60)^d^
Believes you can do something if experience abuse (1=yes, 0=no, n=989 [100%])	0.65 (0.27-1.59)	0.68 (0.28-1.62)
GBV^e^ (1=high/moderate, 0=low, n=701 [70.9%])	0.81 (0.25-2.59)	0.86 (0.28-2.68)

^a^OR: odds ratio.

^b^AOR: adjusted odds ratio.

^c^Indicates a significant difference between intervention and control arms at endline (*P*<.05).

^d^Indicates a significant difference between intervention and control arms at endline (*P*<.01).

^e^GBV: gender-based violence.

Outreach worker contact increased from 19.7% (43/218) at baseline to 31.9% (53/166) at endline in the intervention group and declined from 21.2% (36/170) at baseline to 14.9% (7/47) at endline in the control group. The differences were significant at midline but not at endline. Similarly, escorted referrals increased from 10.6% (23/218) at baseline to 25.9% (43/166) at endline in the intervention group and declined from 13.5% (23/170) at baseline to 6.4% (3/47) at endline in the control group. The differences in escorted referrals were significant at both midline and endline. The proportion of FEWs who reported no forced drinking at work in the past 3 months increased from 56.4% (123/218) at baseline to 70.5% (117/166) at endline in the intervention group and declined from 65.3% (111/170) at baseline to 55.3% (26/47).

**Figure 2:**
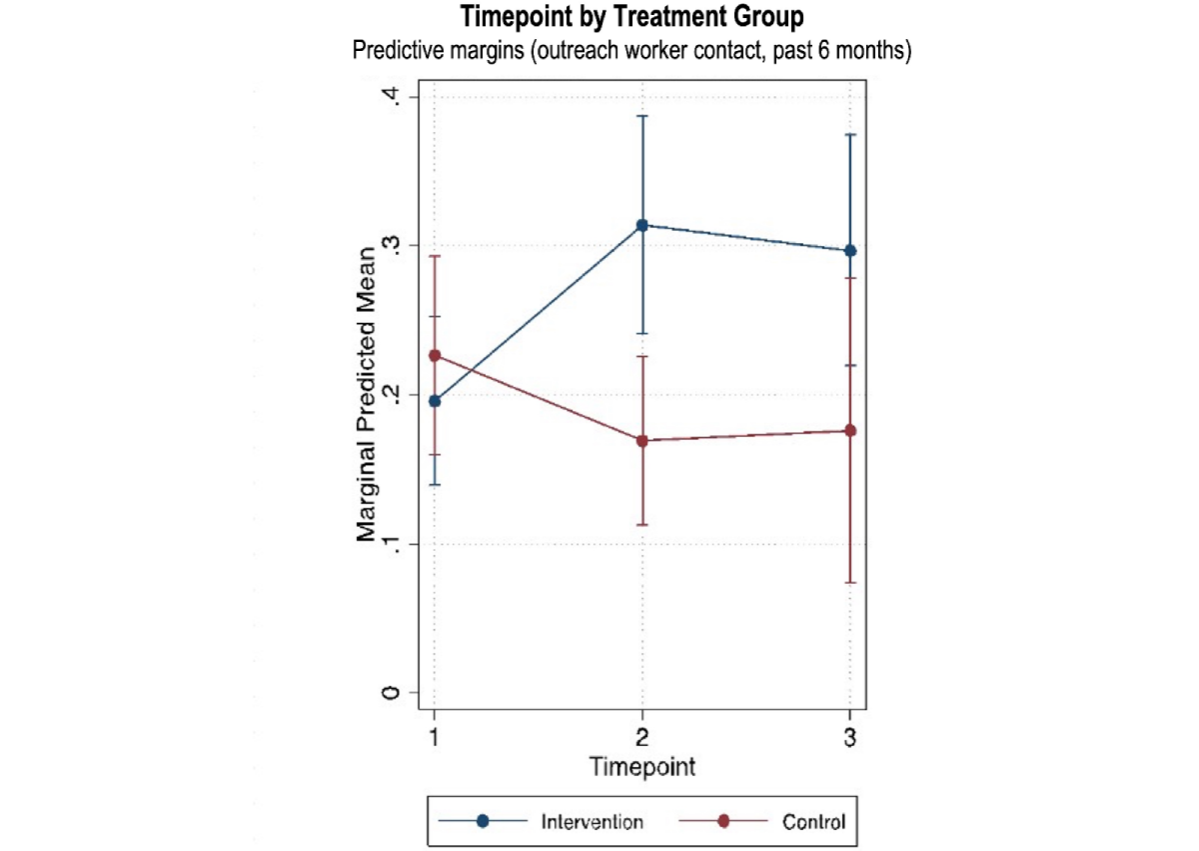
Intervention effects on outreach worker contact by timepoint.

**Figure 3:**
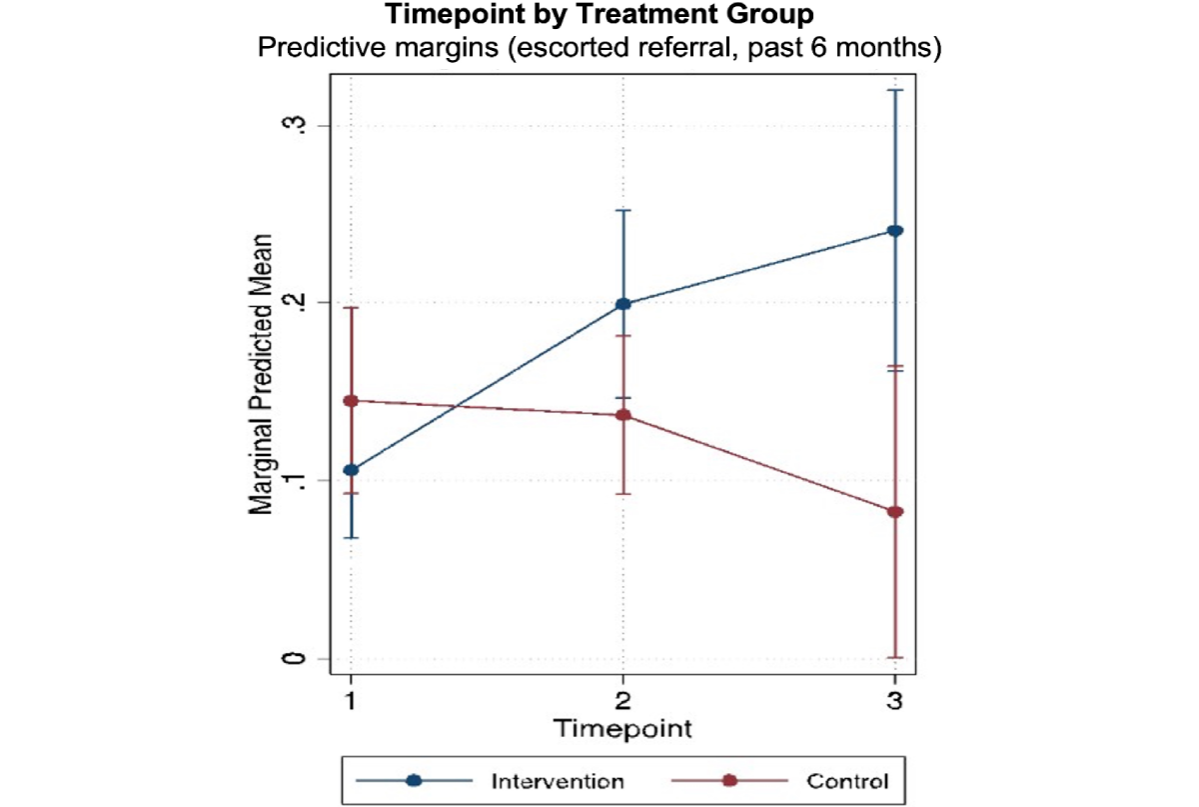
Intervention effects on escorted referrals by timepoint.

**Figure 4:**
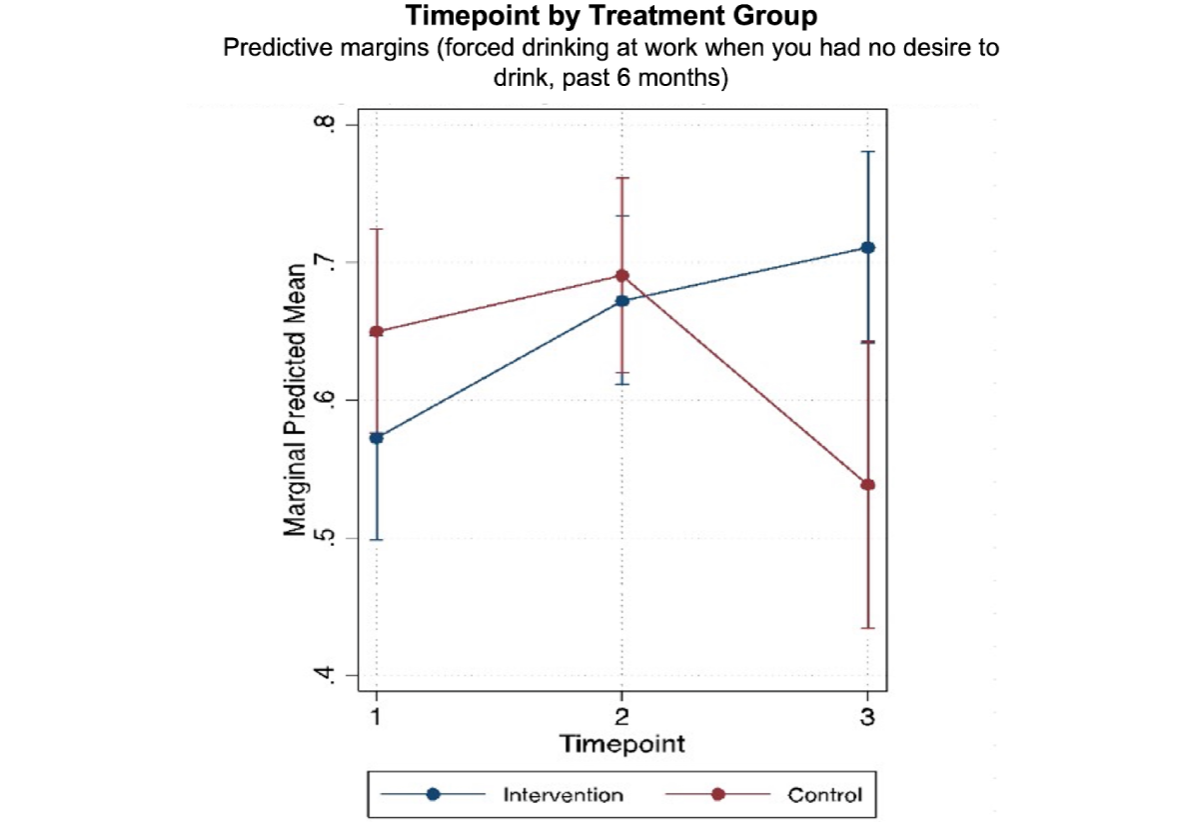
Intervention effects on never being forced to drink at work by timepoint.

Figures 2-4 show significant intervention effects over time in the adjusted models for contact with outreach workers (Figure 2), escorted referrals (Figure 3), and never being forced to drink at work (Figure 4).

## Discussion

### Principal Results

Our findings suggest that the Mobile Link intervention effectively connects FEWs with outreach workers for health information and escorted referrals. Outreach worker contact increased by 61% in the intervention group and decreased by 30% in the control group. Similarly, escorted referrals increased by 144% in the intervention group and decreased by 53% in the control group over the same period. The reductions in forced drinking at work were larger in the intervention group than in the control group. However, the findings do not indicate an impact on the primary outcome measures, including HIV and STI testing, condom use, and contraceptive use. In addition, improvements on many primary and secondary outcomes in both groups might suggest that enrollment in the study had positive effects on FEWs overall, regardless of whether they were assigned to receive intervention messages.

Our findings indicate that the Mobile Link intervention could reduce the prevalence of forced drinking at work. The proportion of FEWs who reported no forced drinking at work in the past 3 months increased by 25% in the intervention group and decreased by 15% in the control group over the same period. The pressure to drink alcohol at work from supervisors and peers is common for FEWs in Cambodia [25]. During our qualitative assessment for intervention development, FEWs expressed a desire to reduce alcohol consumption at work despite this pressure. Messages developed in response to this request and sent out through the Mobile Link platform advised about subtly avoiding and reducing the effect of heavy drinking. The advices included eating large meals before work, drinking lots of water in between drinks, adding lots of ice to displace the alcohol, and sharing tips to reduce pressure to drink more for tips. These suggestions did not rely on structural-level changes and, therefore, may have been more successfully implemented. As alcohol use is linked to increased sexual risk taking and violence, these findings are promising.

Connecting hard-to-reach populations to prevention and treatment services through outreach workers using mobile phone messages has been shown to be effective in other mHealth studies in low- and middle-income countries [27-29]. RCT evaluations of mHealth interventions have demonstrated positive changes in knowledge and attitudes and, as with our study, have been limited in their ability to show changes in health outcomes [29]. In our theory of change, more health knowledge, paired with more contact with outreach workers, will eventually lead to increased health service use and ultimately improved health outcomes. Our study did not detect changes in health outcomes, perhaps because these changes take longer to occur. It is also possible that several trial implementation challenges may have limited our ability to detect health outcomes changes, including the high loss to follow-up, which was identified as an issue for other mHealth studies in Cambodia [30-32].

### Limitations

These findings should be interpreted with regard to study limitations. First, differential loss to follow-up was observed between intervention and control groups, and attrition was exceptionally high among control and replacement participants. This attrition also resulted in a lower overall sample that is underpowered. Second, recruiting new participants to replace those lost to follow-up was conducted according to the initially intended distribution of participants per province rather than where participants were lost, resulting in an overrepresentation of control participants in smaller provinces. Third, high levels of movement between venues among FEWs led to a protocol change, resulting in individual-level rather than venue-level sampling and nonrandom assignment to intervention and control groups. Finally, the participants were not blinded to the intervention. As such, the findings should be interpreted with caution. However, a balance between study arms at baseline was achieved on all primary and secondary outcomes, in both the analytic sample and the full sample, suggesting that intervention and control participants were appropriately matched based on participant characteristics and health behaviors.

### Comparison With Prior Work

Despite these limitations, this study demonstrates that mHealth approaches can link hard-to-reach populations to necessary health services. Similar to the implications of meta-analysis findings of SMS interventions for antiretroviral adherence support in sub-Saharan Africa [19], messaging content may not have significant impacts on behaviors relative to linkage to health care workers (ie, nurses or community health workers). However, other text-messaging intervention RCTs focused heavily on message content and with greater intensity but shorter duration than this intervention found significant impacts on multiple HIV-related risk behaviors and relative to live messaging communication with community health workers [31]. Client-centered messaging content may also enhance and sustain engagement in the intervention and seed motivation to connect to a community health worker for support and escorted service linkages. Effects likely depend on the population’s risk, resources in context, the intervention intensity, and other intervention details.

### Conclusion

Given the positive findings on service linkages for this intervention, we will consider using the Mobile Link model with other key populations in Cambodia and the region. The traditional in-person visitation model by community health workers on 2-week or monthly rotations, the standard of care during this trial, may be enhanced by interventions such as Mobile Link*.* This study is unique because of the extensive qualitative intervention development period. Replication of any messaging service would benefit from qualitative research to inform adaptation. Successfully linking vulnerable young women to outreach workers and escorted referral services through their mobile phones may lead to linkages to other types of services, events, rights-based information, and behavior change messaging. Longer-term messaging and prompts of community health worker linkage have the potential to increase access to services and may impact FEWs’ health outcomes in the future.

## Appendix

Multimedia Appendix 1 CONSORT-eHEALTH checklist (V 1.6.1).

## Abbreviations

AIC: Akaike information criterion
AOR: adjusted odds ratio
BIC: Bayesian information criterion
FEW: female entertainment worker
FGD: focus group discussion
GBV: gender-based violence
HIV: human immunodeficiency virus
IDI: in-depth interview
ITT: intention-to-treat
KII: key informant interview
mHealth: mobile health
NGO: nongovernment organization
RCT: randomized controlled trial
SIM: subscriber identification module
SMS: Short Message Service
SRH: sexual and reproductive health
STI: sexually transmitted infection
VM: voice message
WMC: Women’s Media Center
